# Continuous low-pressure saline perfusion for gastric endoscopic submucosal dissection

**DOI:** 10.1055/a-2427-9429

**Published:** 2024-10-18

**Authors:** Yuji Ino, Hisashi Fukuda, Takashi Ueno, Hiroki Hayashi, Yoshie Nomoto, Haruo Takahashi, Hironori Yamamoto

**Affiliations:** 1 12838 Department of Medicine, Division of Gastroenterology, Jichi Medical University, Shimotsuke, Japan

A method for conducting endoscopic submucosal dissection (ESD) by employing saline infusions in place of carbon dioxide has been reported 1 . This method effectively enhances the endoscopic visual field and maintains optimal intragastric pressure 2 3 . Nevertheless, several limitations are noteworthy. First, blood admixture from bleeding events can significantly compromise visibility. Addressing this issue necessitates removing the incision device to suction the contaminated physiological saline solution. Second, potential bubble formation may obstruct the visual field by getting into the tip attachment. Consequently, operators are required to intermittently activate the foot pump of the water jet to disperse these bubbles.

To mitigate these problems, we employ continuous low-pressure saline perfusion. After inserting the nasogastric tube and securing it to the gastric wall with clips ( Fig. 1 ), an assistant operates the pedal of the water jet pump to continuously irrigate the gastric cavity with low-pressure physiological saline solution ( Fig. 2 ). The advantages of this technique are described in the following.

**Fig. 1:**
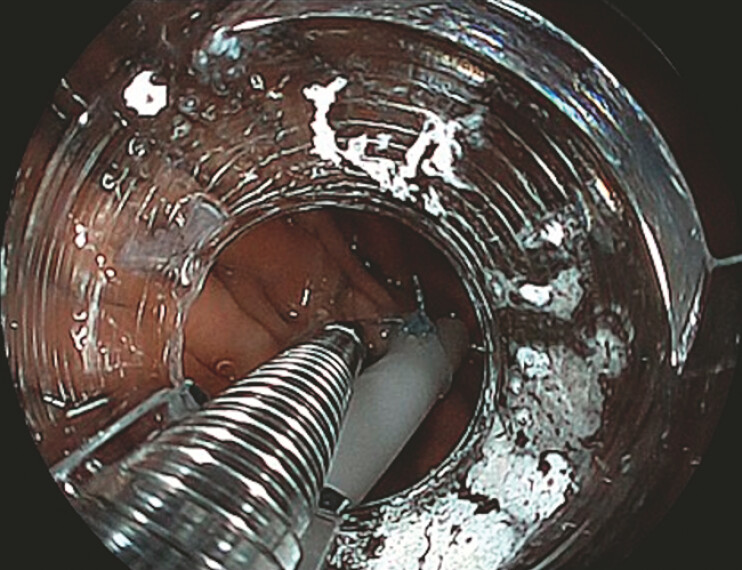
Attach a nasogastric tube with a nylon thread at the tip to the stomach wall using a clip.

**Fig. 2:**
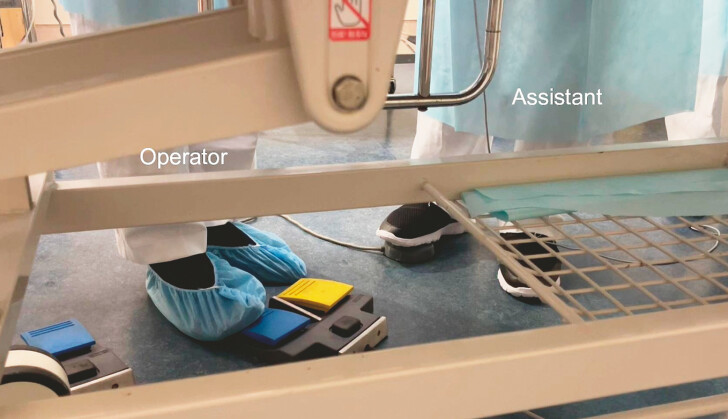
The assistant steps on the water jet pedal, injecting water to create a perfused state.

Sustained irrigation of the interior of the tip attachment with positive pressure facilitates bubble expulsion, thereby ensuring uninterrupted clarity of the visual field ( Fig. 3 , Fig. 4 , Fig. 5 ). Moreover, operators can allocate their attention to manipulating the high-frequency device pedal without needing to engage the foot pedal of the water jet ( Video 1 ). Furthermore, the availability of physiological saline solution retrieval from the nasogastric tube underscores its potential to mitigate hypernatremia and water intoxication, thereby enhancing overall procedural safety 4 . Apart from the standard ESD equipment, the sole requisites for this approach are physiological saline solution and a gastric tube, rendering it remarkably cost-effective.

**Fig. 3:**
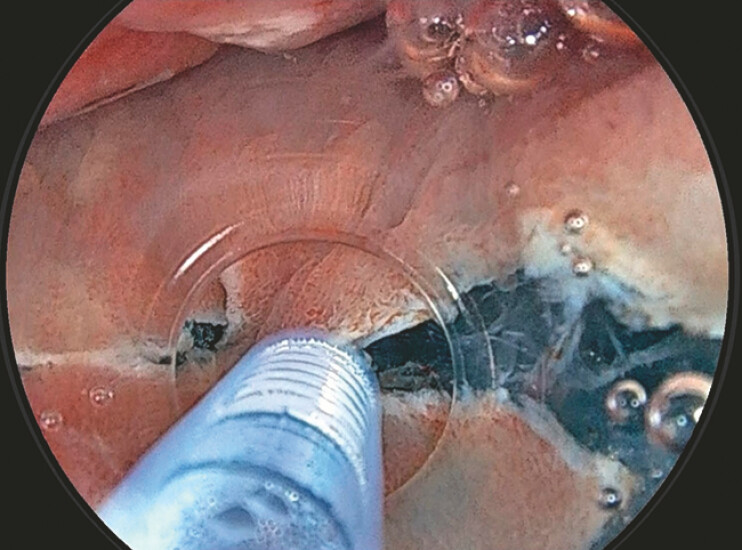
Tiny bubbles appear, but since the inside of the hood is under positive pressure, the bubbles dissipate immediately.

**Fig. 4:**
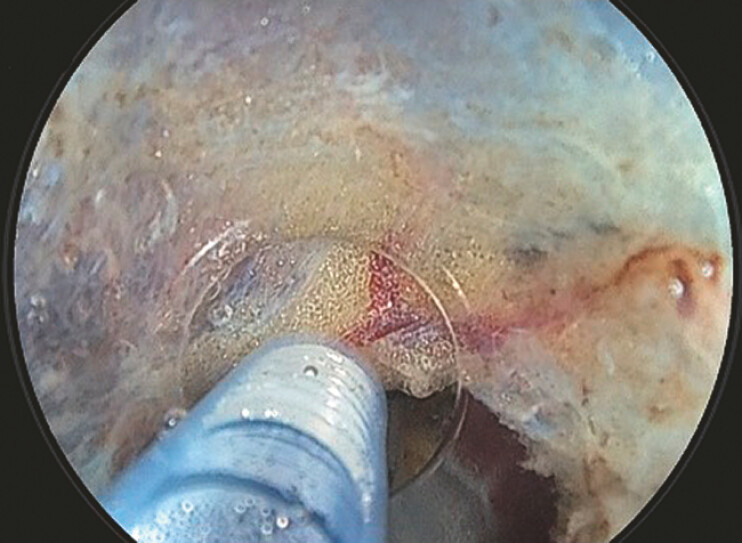
Despite the submucosal layer containing a significant amount of fat, continuous saline perfusion allowed for a clear field of view.

**Fig. 5:**
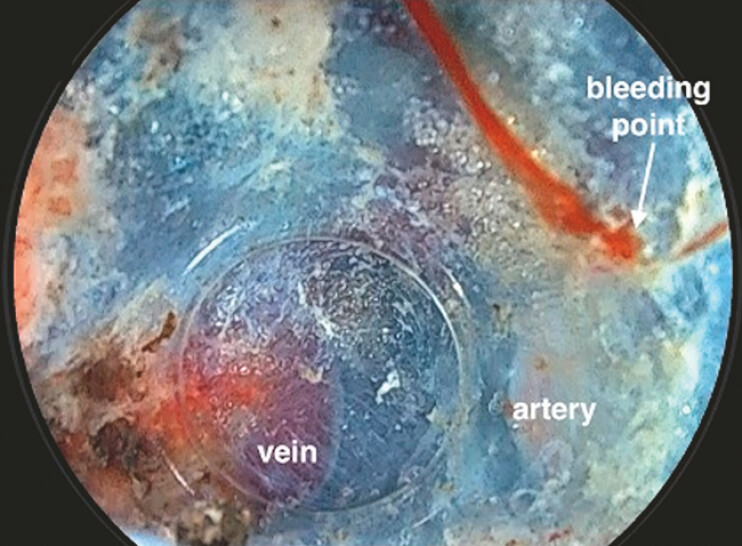
In case of minor bleeding, pressing the bleeding point with the hood can maintain a clear view.

Gastric endoscopic submucosal dissection with continuous low-pressure saline perfusion.Video 1

Endoscopy_UCTN_Code_TTT_1AO_2AG_3AZ

## Notes

Endoscopy E-Videos https://eref.thieme.de/e-videos
*E-Videos* is an open access online section of the journal *Endoscopy* , reporting on interesting cases and new techniques in gastroenterological endoscopy. All papers include a high-quality video and are published with a Creative Commons CC-BY license. Endoscopy E-Videos qualify for HINARI discounts and waivers and eligibility is automatically checked during the submission process. We grant 100% waivers to articles whose corresponding authors are based in Group A countries and 50% waivers to those who are based in Group B countries as classified by Research4Life (see: https://www.research4life.org/access/eligibility/ ). This section has its own submission website at https://mc.manuscriptcentral.com/e-videos .
